# Sustained Immune Complex-Mediated Reduction in CD16 Expression after Vaccination Regulates NK Cell Function

**DOI:** 10.3389/fimmu.2016.00384

**Published:** 2016-09-26

**Authors:** Martin R. Goodier, Chiara Lusa, Sam Sherratt, Ana Rodriguez-Galan, Ron Behrens, Eleanor M. Riley

**Affiliations:** ^1^Department of Immunology and Infection, London School of Hygiene and Tropical Medicine, London, UK; ^2^Department of Clinical Research, London School of Hygiene and Tropical Medicine, London, UK

**Keywords:** NK cells, CD16, CD57, degranulation, CD25, vaccination, influenza

## Abstract

Cross-linking of FcγRIII (CD16) by immune complexes induces antibody-dependent cellular cytotoxicity (ADCC) by natural killer (NK) cells, contributing to control of intracellular pathogens; this pathway can also be targeted for immunotherapy of cancerous or otherwise diseased cells. However, downregulation of CD16 expression on activated NK cells may limit or regulate this response. Here, we report sustained downregulation of CD16 expression on NK cells *in vivo* after intramuscular (but not intranasal) influenza vaccination. CD16 downregulation persisted for at least 12 weeks after vaccination and was associated with robust enhancement of influenza-specific plasma antibodies after intramuscular (but not intranasal) vaccination. This effect could be emulated *in vitro* by co-culture of NK cells with influenza antigen and immune serum and, consistent with the sustained effects after vaccination, only very limited recovery of CD16 expression was observed during long-term *in vitro* culture of immune complex-treated cells. CD16 downregulation was most marked among normally CD16^high^ CD57^+^ NK cells, irrespective of NKG2C expression, and was strongly positively associated with degranulation (surface CD107a expression). CD16 downregulation was partially reversed by inhibition of ADAM17 matrix metalloprotease, leading to a sustained increase in both CD107a and CD25 (IL-2Rα) expression. Both the degranulation and CD25 responses of CD57+ NK cells were uniquely dependent on trivalent influenza vaccine-specific IgG. These data support a role for CD16 in early activation of NK cells after vaccination and for CD16 downregulation as a means to modulate NK cell responses and maintain immune homeostasis of both antibody and T cell-dependent pathways.

## Introduction

Natural killer (NK) cell effector function can be augmented by vaccination as a result of antibody-dependent cellular cytotoxicity (ADCC) and CD4^+^ T cell/IL-2-driven cytokine secretion (1–4). Moreover, we have recently observed that vaccination induces intrinsic changes in NK cell function, resulting in enhanced responsiveness to innate cytokines that may synergize with adaptive immunity to further potentiate NK cell responses (3).

Natural killer cell-mediated ADCC plays an important role in the control of infections and cancers (5, 6). NK cells express a number of immunoglobulin Fc receptors, with high expression of the activating receptor FcγRIIIA (CD16) and low expression of FCγRII (CD32) being a key feature of CD56^dim^ NK cell populations (7, 8). Cross-linking of CD16 by IgG bound to target cell surface antigens leads to degranulation and release of perforin and granzymes. One particular subset of NK cells, characterized by low expression of CD56 but high levels of expression of CD16, CD57, and CD94/NKG2C, has recently been reported to be particularly efficient at mediating ADCC and may indeed be highly specialized for this particular effector function (9, 10). Furthermore, adaptive expansions of NK cells from HCMV-infected individuals have high frequencies of FcεRγ1^−^, PLZF^−^ NK cells with potent ADCC activity against virus-infected target cells (11, 12).

Regulation of NK cell ADCC is achieved in part by the relative strength of signals transduced through activating Fc receptors and NK cell inhibitory receptors but may also rely on matrix-metalloproteinase-9 (MMP9) or ADAM17-mediated cleavage of CD16 from the cell surface after cross-linking by IgG (13–15). For example, rituximab-mediated targeting of CD20^+^ tumor cells results in potent downregulation of NK cell CD16 that is dependent on MMP9-mediated cleavage (13, 14); exposure of NK cells to HCMV-infected fibroblasts leads to loss of CD16 concurrent with increased degranulation (9); MMP-dependent downregulation of CD16 is a feature of chronic HIV-1 infection (16, 17); and soluble NK cell-derived CD16 is elevated during rheumatoid arthritis, a chronic immune complex-associated inflammatory disease (18). However, similar effects have also been reported among cytokine-activated NK cells (stimulated *in vitro* with IL-2, IL-12, and IL-18) (19–21), suggesting that cross-linking of CD16 may not be essential for its downregulation. Importantly, neither the kinetics of CD16 expression after cross-linking nor the functional consequences of CD16 downregulation have been explored in any depth.

Here, we have investigated CD16 expression by NK cells from healthy subjects and find that CD16 is downregulated for many weeks after influenza vaccination, that CD56^dim^ CD57^+^ NK cells are particularly prone to losing CD16 after vaccination, and that this is mediated by vaccine antigen–antibody complexes. Furthermore, we show that ADAM-17 inhibitors or blocking antibodies to ADAM-17 prevent shedding of CD16 in response to vaccine antigens and that sustained CD16 signaling potentiates NK cell degranulation and CD25 expression. These data support a role for CD16 downregulation in regulating NK cell responses *in vivo* and maintaining homeostasis of both antibody and T cell-dependent pathways of NK cell activation.

## Materials and Methods

### Subject Recruitment and Sample Collection

Venous blood was taken from a total of 47 healthy volunteers. The precise number of study subjects for each experiment is stated in the respective figure legends. The impact of recent vaccination on NK cells was studied in 37 healthy adult volunteers (median age 37.5 years; range of 21–63 years). None of the subjects had been previously vaccinated against influenza and none had experienced influenza-like symptoms during the previous 6 months. Subjects were randomly assigned to receive a single dose of 2012–2013 seasonal trivalent influenza vaccine (TIV) by either the intramuscular (Split Virion BP, Sanofi Pasteur MSD) or intranasal (Fluenz, AstraZeneca, UK) route. Randomization was structured so that participants in the two arms of the study could be matched according to age and sex. The intramuscular vaccine contains chemically inactivated virus, while the intranasal vaccine contains live attenuated virus. The vaccines were preservative free and were not adjuvanted. Venous blood samples were obtained immediately prior to vaccination and then at 2, 4, 12, and up to 36 weeks after vaccination. The study was approved by the ethical review committee of the London School of Hygiene and Tropical Medicine (Ref 6237). Locally recruited volunteers participating in influenza vaccination studies were provided with a participant information sheet detailing the studies. All participating volunteers provided written consent. The study made use of fully licensed vaccines which are routinely used in clinical practice. The study Clinician (Dr. Behrens) provided medical supervision for all procedures during the baseline visit and was available for emergencies during subsequent visits and was on hand to provide follow-up care for volunteers who experience side effects of the procedures.

Plasma was stored for assay of antibodies to influenza and for use in autologous cell cultures. PBMC were separated by standard Histopaque (Sigma, UK) gradient centrifugation and stimulated within 3 h of blood collection (for immediate culture experiments) or cryopreserved at 1 × 10^7^ cells/ml in RPMI 1640, 40% fetal calf serum (FCS), 10% DMSO (Sigma, UK), within 4 h of blood collection. Cells were stored for 18 h at –80°C in Nalgene™ cryoboxes with isopropanol coolant prior to transfer to liquid nitrogen for longer term storage (22, 23).

### Cell Culture Conditions, NK Cell Activation

For each individual, cells collected at baseline and at each post-vaccination time point were tested side-by-side. Cryopreserved PBMC were thawed, washed, and counted in Fastread™ counting slides (Immune Systems, UK), as previously described (22, 23), with a median yield of 56% and viability by trypan blue exclusion of 98%. Cells were rested for 4–6 h, in the absence of exogenous cytokines, prior to stimulation. Briefly, 2 × 10^5^ PBMC were cultured for a total of 6 h, or where indicated for 18 h, in culture medium alone or with inactivated TIV (Split Virion BP, Sanofi Pasteur MSD). Cells were also stimulated with high concentrations of cytokines (HCC): IL-12 (5 ng/ml) plus IL-18 (50 ng/ml). For *in vitro* assays, FITC-conjugated anti-CD107a antibody (clone HP9, Beckton Dickinson) was added at the beginning of the culture, according to established protocols (24). GolgiStop (containing Monensin; 1/1500 concentration; BD Biosciences, Oxford, UK) and GolgiPlug (containing Brefeldin A; 1/1000 final concentration; BD Biosciences, Oxford, UK) were added 3 h before the end of the incubation. Assays were performed in 1% pooled AB serum, batch tested for performance in NK cell assays (Sigma, UK) unless otherwise stated. To determine the role of IgG on NK cell responses, pooled AB plasma was depleted of IgG using a protein G sepharose column (Millipore, UK), as previously described (23). *In vitro* neutralization experiments were performed using a rat-anti human IL-2 antibody (Rat IgG2a, clone MQ1-17H12, NA/LE, BD Biosciences) or a rat IgG2a control reagent (eBioscience, UK).

ADAM 17/MMP-dependent cleavage of CD16 was tested using the inhibitor TAPI-1 at a concentration of 10 μM (Merck Millipore, UK) and the active site-specific anti-ADAM17 monoclonal antibody D1 (A12) at a concentration of 6 μg/ml (Millipore, UK) and responses compared with the DMSO vehicle (Sigma, UK) and isotype-matched mouse IgG1 control (eBioscience, UK) treatments. Optimal concentrations of inhibitors were based on published protocols (25, 26) and confirmed by titration. Inhibitors were added 30 min prior to stimuli unless otherwise stated.

Long-term, *in vitro* culture of NK cells was performed after stimulation of PBMC for 18 h and washing cells (three times) to remove stimuli. Cells were maintained in RPMI 1640 supplemented with 5% AB serum and 0.75 ng/ml of IL-15, replacing the medium every 3 days. CD16 expression was monitored by flow cytometric analysis up to 18 days after initial stimulation, as described below.

### Flow Cytometric and ImageStream™ Analysis

Phenotypic and functional analysis of NK cells was performed with the following monoclonal antibodies: anti-CD3-V500 (Clone UCHT1), anti-CD56-PeCy7 (B159), anti-CD107a-FITC (H4A3) (all from BD Biosciences), anti-CD57-e450 (TB01), anti-CD16-APC (CB16), and anti-CD69 PE (all from eBioscience). Dead/apoptotic cells were excluded using APC-efluor780-conjugated fixable viability dye (eBioscience). Cells were acquired on an LSRII flow cytometer (BD Biosciences, Oxford, UK) using FACSDiva^®^ software.

**Figure 1:**
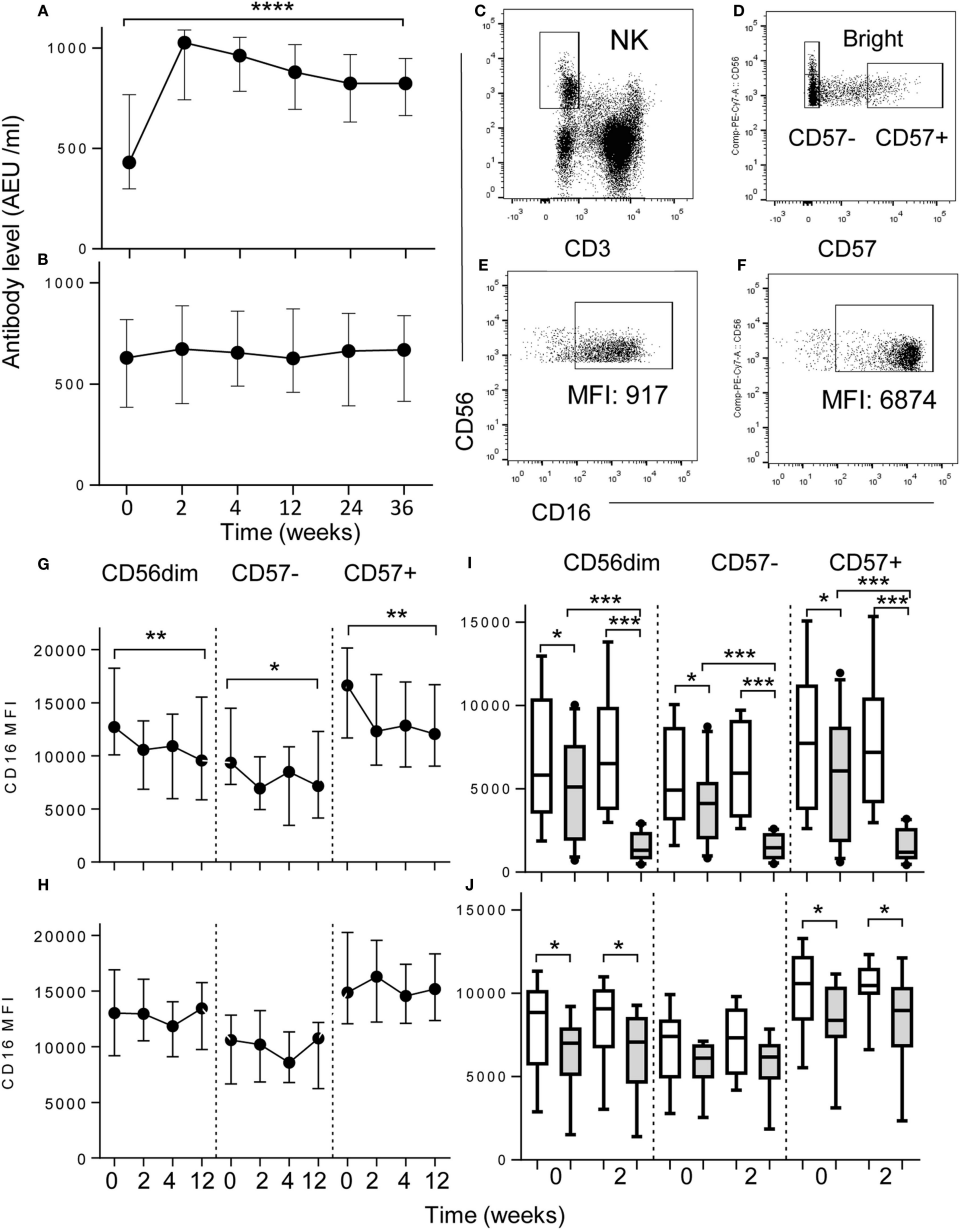
**Downregulation of NK cell CD16 expression after vaccination**. **(A,B)** Increased plasma concentrations of anti-influenza IgG after **(A)** intramuscular (I.M, *n* = 17) but not after **(B)** intranasal vaccination (I. N, *n* = 18) up to 36 weeks after vaccination. Data represent median values with interquartile ranges. **(C–F)** Flow cytometric gating to assess the impact of vaccination on CD16 expression in CD56^dim^, CD57^−^, or CD57^+^ NK cell subsets, *ex vivo*. NK cells were gated as CD56^+^ CD3^−^ **(C)** and then as CD56^bright^, CD56^dim^CD57^−^ or CD56^dim^CD57^+^ **(D)**. **(E,F)** For determination of antigen-driven effects CD16^+^ CD56^+^ NK cells were gated among PBMC cultured with TIV and immune plasma **(E)** or immune plasma without TIV **(F)**. *Ex vivo* analysis of CD16 expression at baseline (0), 2, 4, and 12 weeks after intramuscular (I. M) vaccination with TIV **(G)** or intranasal (I.N.) vaccination with LAIV **(H)**. Impact on NK cell CD16 expression of culturing baseline PBMC with TIV (shaded bars) or without TIV (open bars) and either pre- (0) and post- (2) vaccination I.M. **(I)** or I.N. **(J)** plasma; data are shown for CD56^dim^, CD57^−^, and CD57^+^ NK subsets after 6 h of *in vitro* culture with TIV. Trend analysis was performed using a one-way repeated measures ANOVA. Paired comparisons between pre- and post-vaccination plasma were made using Mann–Whitney *U* test. *p < 0.05, **p < 0.01, ***p < 0.001, ****p < 0.0001.

Data analysis was performed using FlowJo V10 (Tree Star). FACS gates set on unstimulated cells (medium alone or isotype controls) were applied across all samples and all conditions. Responses where the gated subset contained <100 events were excluded. CD57^+^ subsets were gated using an isotype-matched control reagent (mIgG1-eFluor450, eBioscience). Sample gating strategies are shown in Figure 1.

Image-stream™ analysis was performed to test for internalization of CD16 after activation. 2 × 10^6^ freshly isolated PBMC were cultured for a total of 5 h in the presence of antigen and human plasma. FCS was substituted as a negative control for human Ig-containing plasma. After 5 h, cells were labeled with the following monoclonal antibodies for detection of surface antigens: anti-CD3 PE, anti-CD56PeCy7, anti-CD16APC (Clone 3G8, Biolegend), and anti-CD57e450 and were blocked for an additional 15 min with unconjugated anti-CD16 (Clone 3G8, BD Biosciences) antibody to ensure occupation of all relevant epitopes prior to intracellular staining. After fixation and permeabilzation [Fix-Perm kit (BD Biosciences)], cells were incubated for a further 30 min with anti-CD16 Pe-Dazzle 594 (Clone 3G8, Biolegend) to detect any internalized CD16. PBMC were then washed in permwash buffer (BD Biosciences) and resuspended in PBS at a minimum concentration of 2 × 10^7^/ml. Events were acquired on an ImageStream^®^ X Mark II Imaging Flow Cytometer (Amnis^®^) using the associated INSPIRE^®^ software for acquisition and IDEAS^®^ software for analysis. FACS gates were set based on FCS controls and applied across all samples and conditions. CD56^+^ CD3^−^ NK cells were inspected visually to determine the presence or absence of internalized CD16.

### Statistical Analysis

Statistical analysis was performed using GraphPad Prism version 6.02. Linear trends were evaluated using repeated measures ANOVA. Functional responses between different culture conditions or between vaccination time points were compared using Wilcoxon signed-rank test. Significance levels are assigned as *p < 0.05, **p < 0.01, ***p < 0.001, and ****p < 0.0001 for all tests.

## Results

### CD16 Is Downregulated on NK Cells after Intramuscular Influenza Vaccination and Is Antibody Dependent

Intramuscular vaccination with inactivated, trivalent seasonal influenza vaccine (TIV) significantly enhanced plasma concentrations of influenza-specific IgG up to 36 weeks after vaccination (Figure 1A) whereas little or no change in systemic IgG concentrations was seen after intranasal vaccination with the live attenuated influenza vaccine (LAIV) (Figure 1B).

To see whether vaccination affected the phenotype of peripheral blood NK cells, PBMC collected before vaccination and 2, 4, and 12 weeks after vaccination were stained immediately *ex vivo* for surface expression of CD56, CD16 and, as a marker of NK cell maturity, CD57. Examples of the flow cytometric gating strategy are shown in Figures 1C–F. Among those vaccinated intramuscularly with TIV, CD16 expression (MFI) on CD56^dim^ NK cells was significantly reduced 2 weeks after vaccination in comparison to the pre-vaccination (baseline level) (Figure 1G); this was sustained for at least 12 weeks post-vaccination and was most pronounced among the CD57^+^ subset. In complete contrast, there was no significant change in CD16 expression on NK cells of those vaccinated intra-nasally with LAIV (Figure 1H).

As downregulation of CD16 correlated with induction of anti-influenza antibodies, we hypothesized that these two observations were causally linked. To determine whether anti-influenza IgG contributed to CD16 downregulation, we cultured PBMC (collected at baseline, 0 weeks) with or without TIV antigens in the presence of autologous plasma collected at baseline (0 weeks) or after vaccination (2 weeks) (Figures 1I,J). Significant downregulation of CD16 was observed in cells cultured with autologous week 0 plasma and TIV antigen compared to without antigen, presumably reflecting the presence of pre-existing, influenza-specific antibodies resulting from prior infection (Figures 1I,J). However, when cells were cultured with TIV and 2 weeks post-vaccination plasma from TIV-vaccinated donors, there was a pronounced further reduction in CD16 expression on both CD57^−^ and CD57^+^ NK cells compared to cells cultured without antigen (Figure 1I) but no such effect was observed with post-vaccination plasma from LAIV-vaccinated donors (Figure 1J). Furthermore, for TIV-vaccinated donors, the extent of CD16 downregulation was dependent on plasma concentration, and was seen in both CD57^−^ and CD57^+^ NK cells and in both NKG2C^+^ and NKG2C^−^ NK cell subsets (Figures S1A,B Supplementary Material). Together, these data indicate that downregulation of CD16 is a sensitive indicator of vaccine-induced IgG concentration.

### Downregulation of CD16 on NK Cells Is Due to CD16 Shedding and Is Slow to Recover

**Figure 2:**
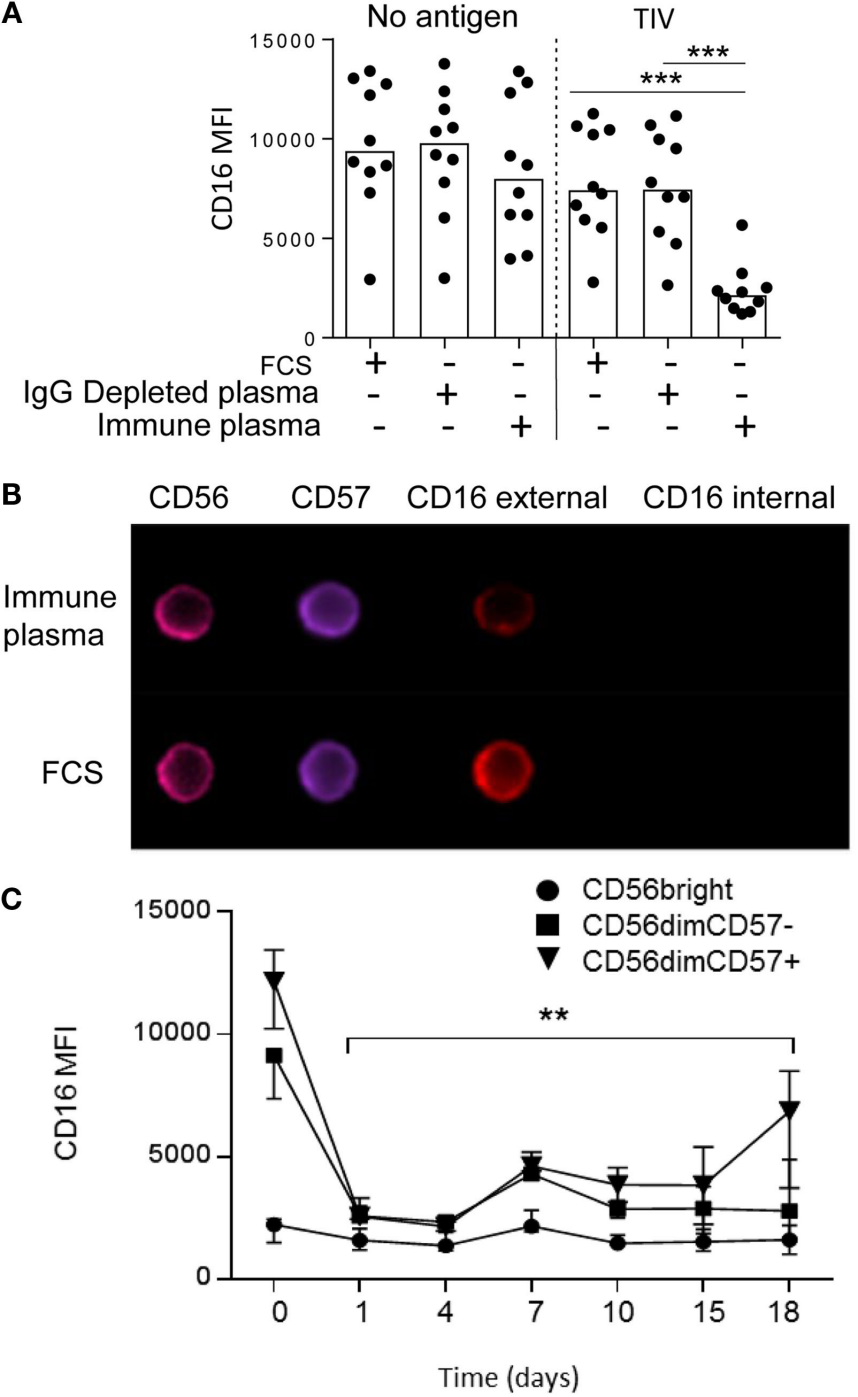
**Immune complex-induced shedding and sustained downregulation of NK cell CD16 expression**. **(A)** Downregulation of CD16 requires IgG-replete human plasma and TIV antigen. PBMC were cultured for 5 h in FCS, IgG-depleted human plasma or IgG-replete plasma in the presence or absence of TIV. **(B)** ImageStream™ analysis of NK cells after 5 h culture with TIV plus human plasma (containing anti-influenza IgG) or FCS. Examples are shown of CD56^dim^ CD57^+^ NK cells stained for surface (external) and intracellular (internal) CD16. **(C)** Time course for recovery of CD16 expression *in vitro*. Expression of CD16 was tracked by flow cytometry up to 18 days after culture of PBMC from five separate donors with TIV for 18 h in the presence of 1% human plasma (containing anti-influenza IgG). CD16 expression is shown for CD56^bright^, CD56^dim^CD57^−^, and CD56^dim^ CD57^+^ NK cell subsets. Trend analysis was performed using a one-way repeated measures ANOVA (*p < 0.05).

IgG-dependent downregulation of CD16 may be due to internalization of CD16–IgG–Ag complexes or to cleavage of CD16 at the cell surface and shedding into the extracellular milieu. Downregulation of CD16 did not occur in cells cultured in FCS or in either IgG-depleted or IgG-replete human plasma in the absence of TIV antigen. CD16 downregulation was seen only when cells were cultured in IgG-replete plasma with TIV antigen but not when TIV antigen was added to cultures containing FCS or IgG-depleted plasma (Figure 2A). To determine whether CD16 was internalized after IgG–Ag cross-linking, NK cells were incubated with TIV plus IgG-replete (immune) human plasma or FCS for 5 h and analyzed by Imagestream™ for extracellular and intracellular CD16 (Figure 2B). Surface staining for CD16 was clearly visible on CD56^dim^ CD57^+^ NK cells cultured with TIV + FCS but not on cells cultured with TIV and immune human plasma. In neither case was CD16 detected intracellularly, indicating that loss of CD16 from the cell surface was not accompanied by internalization of intact CD16.

The sustained downregulation of CD16 observed *ex vivo* after TIV vaccination suggested that recovery of CD16 expression may be a slow process. To test this, downregulation of CD16 was induced by culturing PBMC with TIV and autologous plasma for 18 h; after washing to remove unbound antigen and antibody, the cells were maintained in a low concentration of IL-15 for 18 days (Figure 2C). CD16 expression was totally ablated on CD56^dim^ NK cells 24 h after activation and remained low until day 7 when there was a partial but sustained recovery; a further slight increase in CD16 expression was seen among CD57^+^ NK cells between 15 and 18 days. However, CD16 expression remained significantly below baseline levels in both CD57^+^ and CD57^−^ NK cells until at least 18 days after activation. These data, taken together with our *ex vivo* observations, suggest that CD16 downregulation persists for several weeks after exposure to antigen in the presence of specific antibody.

### Downregulation of CD16 Correlates with Degranulation

**Figure 3:**
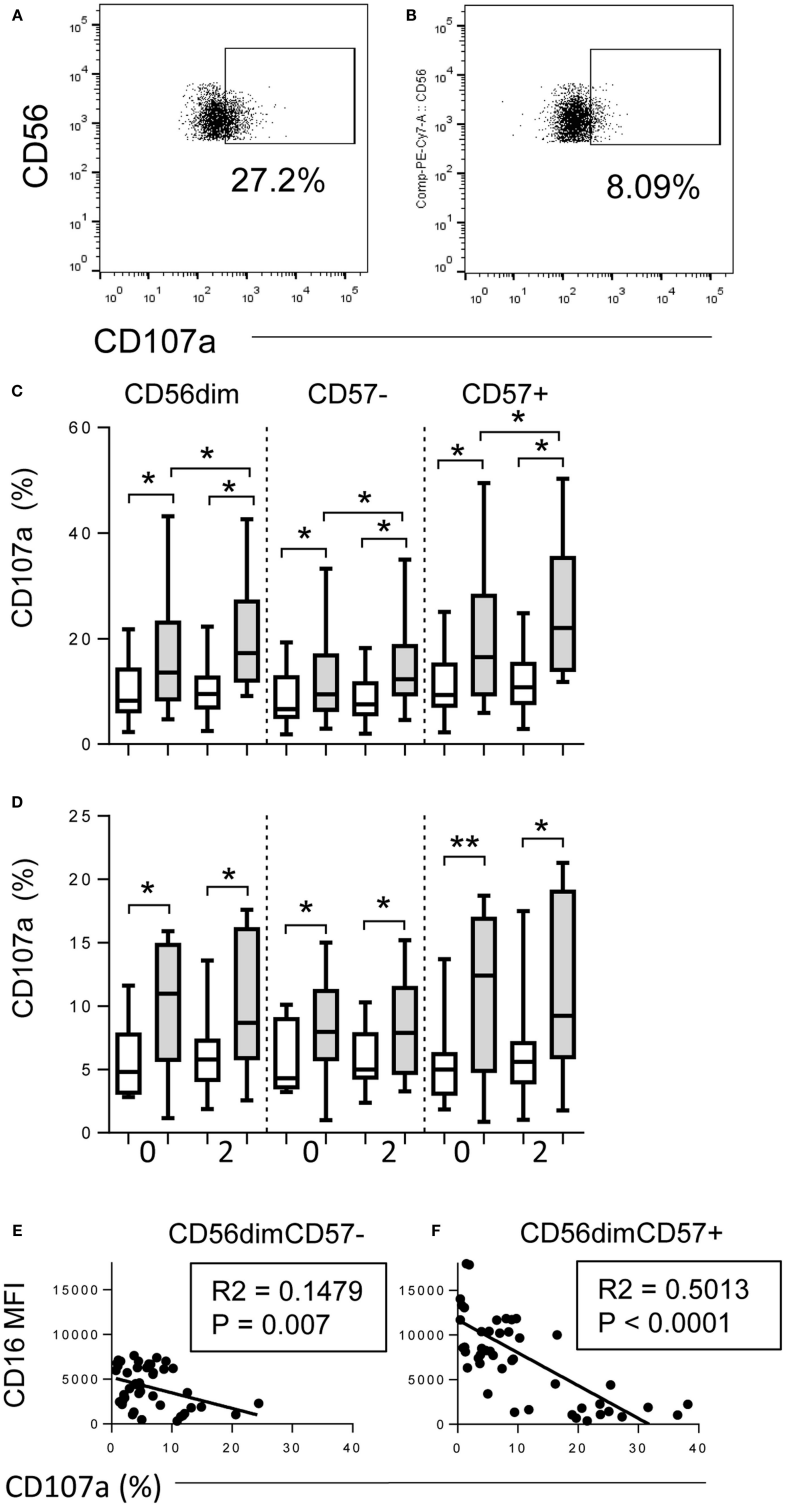
**Downregulation of CD16 correlates with degranulation**. **(A,B)** Flow cytometry gating strategy for CD107a expression on CD56^+^ NK cells cultured with immune plasma **(A)** with or **(B)** without TIV. **(C,D)** Proportions of CD56^dim^, CD56^dim^CD57^−^ and CD56^dim^ CD57^+^ NK cells expressing CD107a after culture of baseline PBMC for 6 h with TIV (shaded bars) or without TIV (open bars) in the presence of baseline (0) or 2 weeks post-vaccination (2) plasma. Individuals (*n* = 10 per group) were vaccinated I.M. with TIV (C) or I.N. with LAIV (D). **(E,F)** Correlation between CD16 expression (MFI) and frequency of CD107a-expressing NK cells within CD57^−^ **(E)** and CD57^+^ **(F)** NK cells after culture for 6 h with TIV and immune plasma. Comparisons between conditions were made using Mann–Whitney *U* test. Correlations were performed using linear regression. *p < 0.05, **p < 0.01, ***p < 0.001.

As CD16 downregulation is IgG–Ag-mediated and, thus, likely linked to ADCC, we explored the relationship between influenza vaccination, NK cell CD16 expression and degranulation (Figure 3). PBMCs were collected at baseline from TIV- and LAIV-vaccinated donors and cultured for 6 h with or without TIV and autologous plasma collected at baseline or 2 weeks after vaccination. Sample plots for analysis of CD107a expression are shown for NK cells cultured in autologous plasma with TIV or, as a negative control, without TIV (Figures 3A,B). Modest, but statistically significant, degranulation was seen in cells cultured with baseline (week 0) plasma for both TIV-vaccinated (Figure 3C) and LAIV-vaccinated (Figure 3D) individuals, again likely reflecting the presence of anti-influenza antibodies due to environmental exposure. Degranulation was, however, further enhanced in the presence of post-vaccination plasma (2 weeks) from TIV-vaccinated individuals (Figure 3C) but not from individuals receiving LAIV (Figure 3D). As expected, degranulation responses were stronger among CD57^+^ NK cells than among CD57^−^ NK cells and CD16 expression was strongly inversely associated with CD107a expression (Figures 3E,F). As expected, for TIV-vaccinated donors, the extent of CD107a expression was dependent upon plasma concentration, and was greater among CD57^+^ NK cells than among CD57^−^ NK cells, although there was no evidence that NKG2C^+^ cells responded more strongly than NKG2C^−^ cells (Supplementary Figure S1 C,D).

### Shedding of CD16 Is Mediated by ADAM17

**Figure 4:**
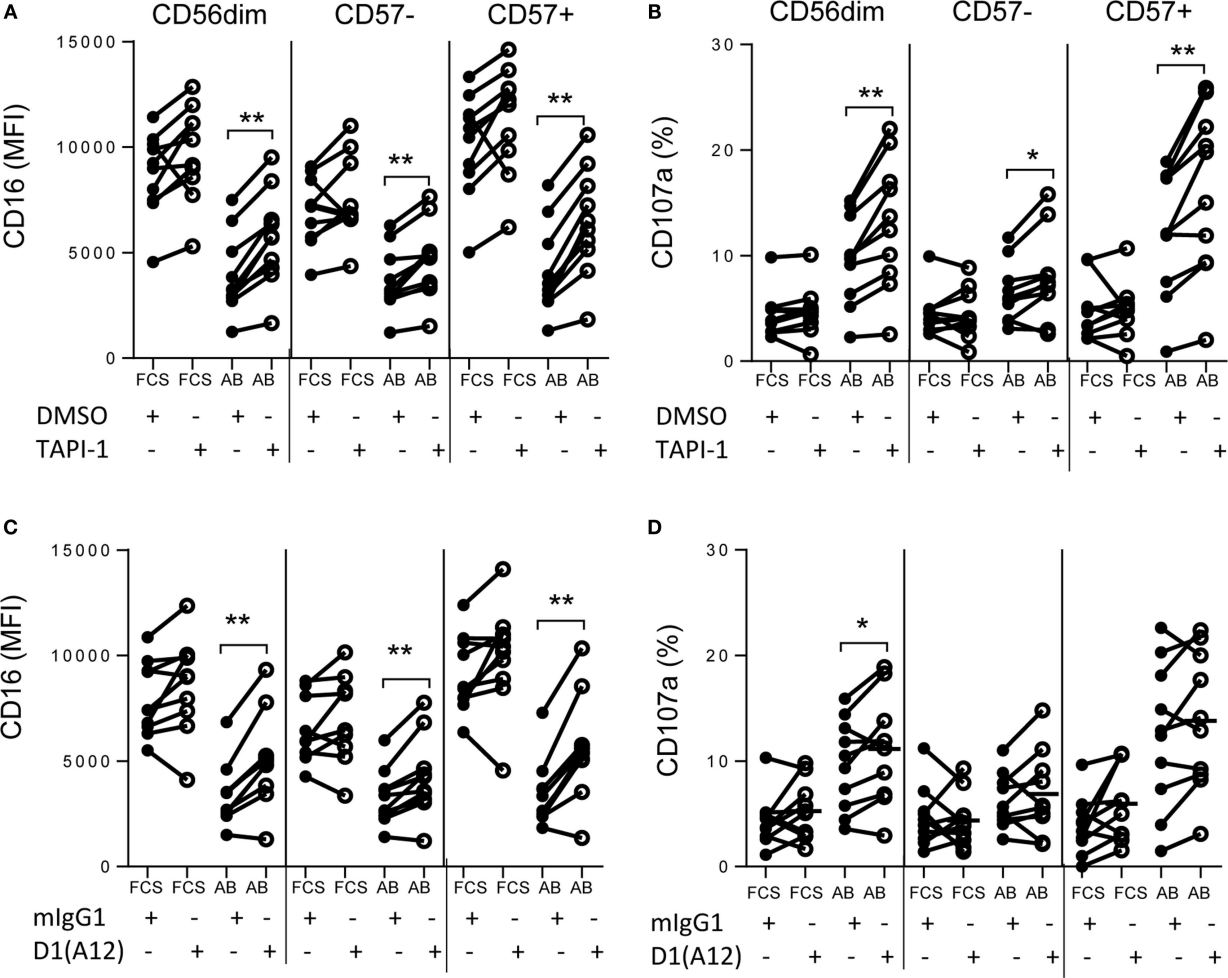
**Shedding of CD16 is mediated by ADAM17**. PBMCs were cultured for 6 h with TIV and FCS or immune AB plasma (AB) in the presence or absence of the MMP inhibitor TAP1-1 **(A,B)** or the D1(A12) blocking antibody to ADAM17 **(C,D)** and the relevant negative controls (DMSO and mIgG1, respectively). CD16 (MFI) **(A,C)** and CD107a (%) **(B,D)** were assessed by flow cytometry on CD56^dim^, CD56^dim^CD57^−^ and CD56^dim^CD57^+^ NK cells. Data are presented for 10 different individuals. Paired comparisons between conditions were made using Mann–Whitney *U* test. *p < 0.05, **p < 0.01, ***p < 0.001.

CD16 shedding was significantly reduced on both CD57^−^ and CD57^+^ NK cells when they were incubated with TIV and immune plasma in the presence of the ADAM17 inhibitor, TAPI-1 (Figure 4A) but not when incubated with the DMSO vehicle control. Notably, TAPI-1-mediated maintenance of CD16 expression was associated with enhanced degranulation as indicated by a significant increase in the frequency of CD107a^+^ cells, particularly within the highly cytotoxic CD56^dim^CD57^+^ subset (Figure 4B). While there is a modest trend toward an increase in CD16 MFI when NK cells are cultured with FCS in the presence of TAPI-1, this is not statistically significant and there is no effect on degranulation. Moreover, blocking the active site of ADAM17 with a specific monoclonal antibody also prevented IgG–TIV-mediated shedding of CD16 (Figure 4C) and enhanced CD107a expression, although to a somewhat lesser extent than the TAPI-1 inhibitor (Figure 4D); these effects were not observed when cells were cultured in FCS rather than immune human serum. However, although specific monoclonal antibody blockade of ADAM17 was sufficient to prevent CD16 shedding, the more effective enhancement of degranulation by the TAPI-1 inhibitor suggests that this may additionally target other molecules involved in the discharge or recycling of cytotoxic granules.

### Early Immune Complex-Mediated Responses Condition Later Events

**Figure 5:**
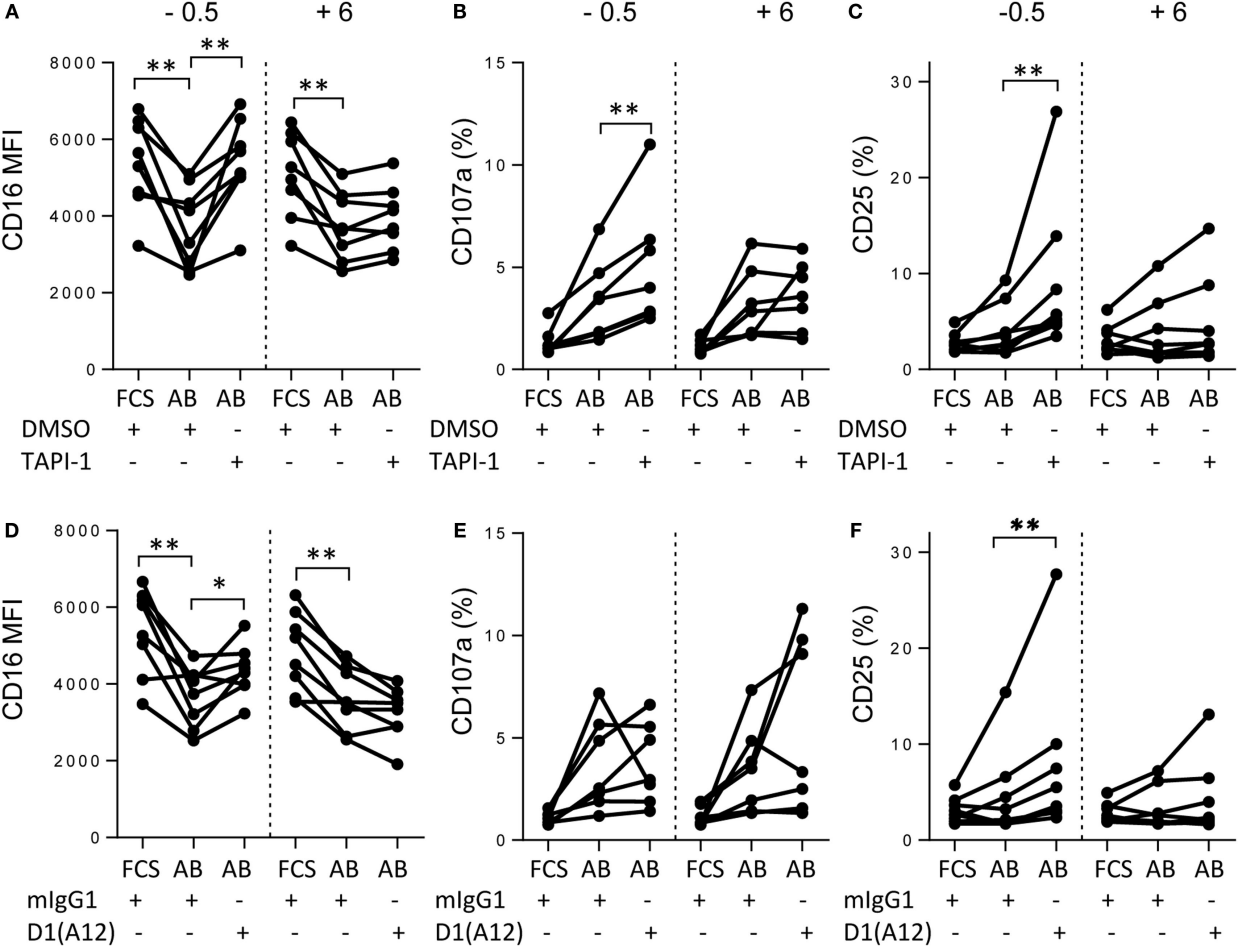
**Early immune complex-mediated responses condition later events**. PBMCs were cultured for a total of 18 h with TIV and FCS or immune AB plasma (AB) in the presence or absence of the MMP inhibitor TAP1-1 **(A–C)** or the D1(A12) blocking antibody to ADAM17 **(D–F)** and the relevant negative controls (DMSO and mIgG1, respectively). CD16 (MFI) **(A,D)**, CD107a (%) **(B,E)**, and CD25 **(C,F)** expression were assessed by flow cytometry. TAPI-1, DMSO, D1(A12), or mIgG1 were added either 30 min prior to (−0.5), or 6 h after (+6) TIV antigen. Data are presented for eight different individuals. Paired comparisons between conditions were made using the Mann–Whitney *U* test. *p < 0.05, **p < 0.01, ***p < 0.001.

Our previous studies have suggested that early, antibody-dependent mechanisms may synergize with antigen-specific T cell responses to enhance NK cell responses to vaccines (3, 23). Since sustained expression of CD16 in the presence of ADAM17/MMP inhibitors led to enhanced degranulation (Figure 5), we investigated whether prevention of CD16 shedding would affect other NK cell responses. ADAM17/MMP blockade had no impact on IgG–TIV-induced CD69 expression, nor was there any impact on CD25 expression after 6 h of culture (Figure S2 in Supplementary Material). However in 18 h cultures, addition of TAPI-1 (Figures 5A–C) or blocking antibody to ADAM17 (Figures 5D–F) 30 min before the start of the culture (time – 0.5 h) not only sustained CD16 expression (Figures 5A,D) and enhanced CD107a expression (Figure 5B) but also significantly enhanced CD25 expression compared to control cultures (Figures 5C,F). However, when addition of TAPI-1 or anti-ADAM17 was delayed for 6 h after the initiation of cultures (+ 6 h) CD16 expression could not be rescued (Figures 5A,D) and there was no enhancement of degranulation or CD25 expression (Figures 5B,C,E,F).

### Bi-directional Cross-talk between CD16-Mediated and Cytokine-Mediated Pathways of NK Cell Activation

**Figure 6:**
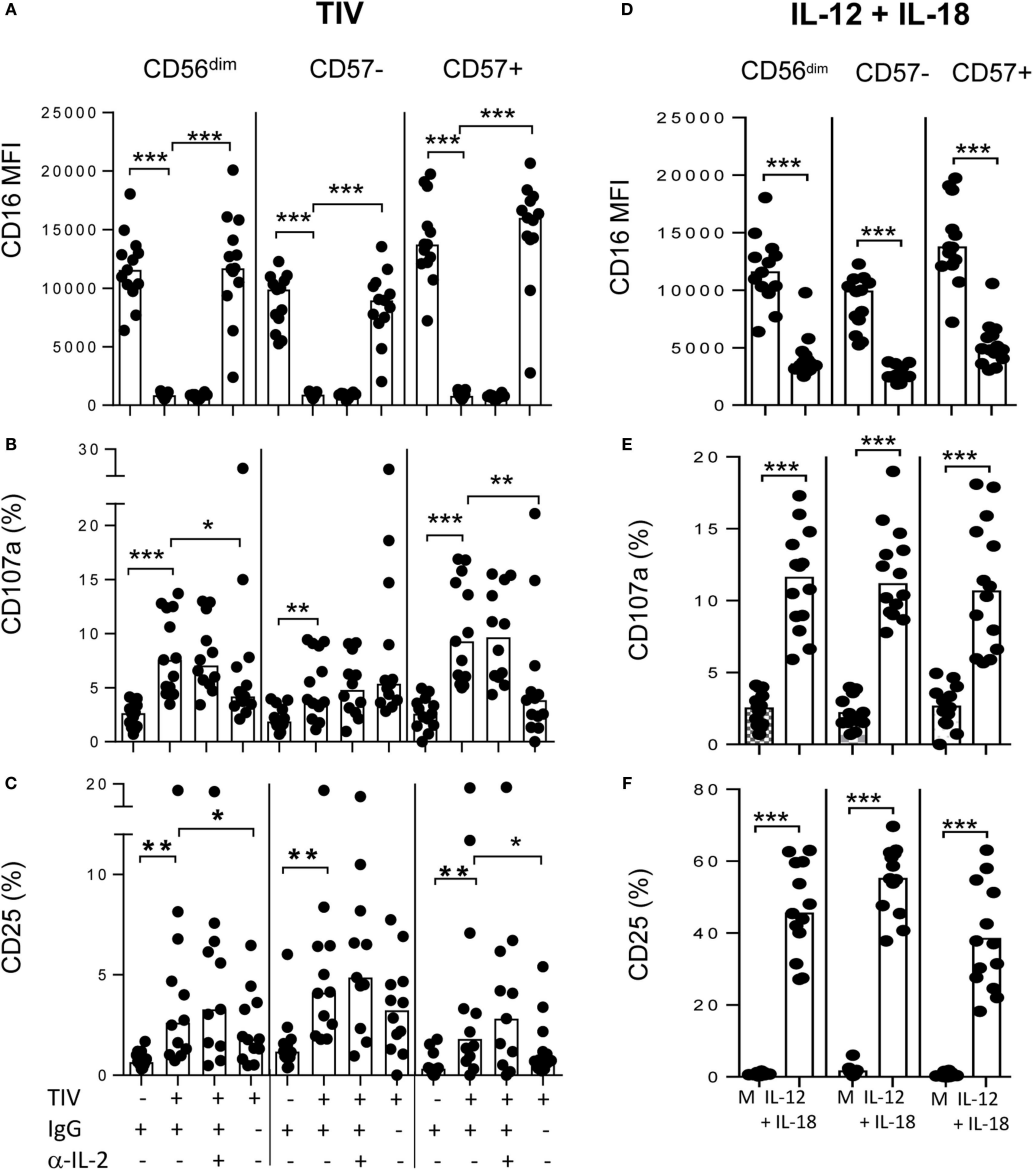
**Bi-directional cross-talk between CD16-mediated and cytokine-mediated pathways of NK cell activation**. **(A–C)** PBMCs were cultured for 18 h with or without TIV in the presence of immune plasma (IgG+) or IgG-depleted immune plasma (IgG−) or after treatment with anti-IL-2 neutralizing antibody or isotype-matched control and expression of CD16 MFI **(A)**, CD107a (%) **(B)**, or CD25 (%) **(C)** on CD56^dim^, CD56^dim^CD57^−^, and CD56^dim^CD57^+^ NK cells was assessed by flow cytometry. **(D–F)** PBMCs were cultured for 18 h with or without IL-12 (5 ng/ml) combined with IL-18 (50 ng/ml) and expression of CD16 MFI **(D)**, CD107a (%) **(E)**, or CD25 (%) **(F)** on CD56^dim^, CD56^dim^CD57^−^, and CD56^dim^CD57^+^ NK cells was assessed by flow cytometry. Paired comparisons were made using the Mann–Whitney *U* test. *p < 0.05, **p < 0.01, ***p < 0.001.

The data presented in Figure 5 are consistent with a role for antigen–antibody complex signaling via CD16 in induction of CD25 as well as in degranulation/cytotoxicity. To explore this further, PBMCs were cultured with TIV for 18 h in the presence of intact immune plasma or immune plasma that had been depleted of IgG by passage over a protein G column (Figure 6). The concentration of anti-TIV–IgG in intact plasma was 413.4 arbitrary ELISA units (AEU)/ml and this was reduced to 7.4 AEU/ml after protein G depletion.

As expected, there was complete loss of CD16 from the surface of NK cells cultured for 18 h with TIV and immune plasma. CD16 shedding was seen in both CD57^−^ and CD57^+^ NK cells and was completely prevented by depleting the immune plasma of IgG (Figure 6A). Also as expected from our previous data, degranulation was inversely correlated with CD16 expression: CD107a expression was induced by TIV plus immune plasma in both CD57^+^ and CD57^−^ subsets (Figure 6B). However, this effect was reversed by culture with IgG-depleted only for CD57^+^ NK cells, consistent with this subset being more dependent on direct activation by TIV–IgG complexes (Figure 6B). In line with observations in Figure 5, TIV plus immune plasma also significantly upregulated CD25 expression on all NK cell subsets (Figure 6C). CD25 upregulation was dependent upon IgG–Ag immune complexes, particularly within CD57+ NK cells, since no induction of CD25 was observed when cells were cultured with TIV and IgG-depleted plasma (Figure 6C).

These observations suggest that there is cross-talk between the CD16 immune complex-mediated pathway and the cytokine-mediated pathway of NK cell activation. To determine whether this cross-talk was bi-directional, CD16, CD107a, and CD25 expression were characterized on PBMCs incubated for 18 h with recombinant IL-12 and IL-18. As shown previously (3, 23, 27), IL-12+IL-18 induces degranulation and strongly upregulates CD25 expression on both CD57^−^ and CD57^+^ NK cells (Figures 6E,F). Importantly, however, IL-12+IL-18 also induced significant shedding of CD16 from the surface of NK cells (Figure 6D), indicating that cytokine-mediated activation of NK cells may restrict their subsequent antibody-dependent responses. Also, as high concentrations of IL-2 have previously been reported to downregulate CD16 (19), and as IL-2 might be being produced in these cultures by TIV-specific T cells, we added neutralizing anti-IL-2 antibodies to our cultures to test whether IL-2 might be contributing to CD16 downregulation. However, neutralization of IL-2 did not affect downregulation of CD16 by TIV/IgG nor the associated induction of CD107a and CD25 (Figures 6A–C).

## Discussion

It is increasingly being recognized that human NK cells are functionally as well as phenotypically heterogeneous, with markers of NK cell differentiation and maturation correlating with effector function. More differentiated NK cells, bearing high levels of CD57 and also expressing CD16, NKG2C, KIR, and the FcεRγ adaptor protein, appear specialized for ADCC, whereas CD57^−^ NK cells (both CD56^bright^ and CD56^dim^ subsets) are highly sensitive to exogenous cytokines. However, NK cell function is not binary: CD57^−^ NK cells express CD16 and can mediate ADCC whereas CD57^+^ NK cells can be induced to secrete IFN-γ under certain circumstances. Moreover, as shown here, immune complex-mediated pathways of NK cell activation influence cytokine-induced pathways, and vice versa.

Natural killer cells are essential for resistance to infection and cancer but – if unregulated – have the potential to cause significant immunopathology (28); NK cell activation is well known, therefore, to be very tightly controlled by the balance of activating and inhibitory signals. However, rather less attention has been paid to regulating the consequences of NK cell activation and restoring immune homeostasis after an infection or other threat has been controlled or eliminated. It is in this context that downregulation of CD16 after cross-linking by immune complexes is likely to be of functional significance.

Internalization, degradation, or shedding of cell surface receptors after ligation by their cognate ligand is a characteristic of the immune system and appears designed to release active soluble mediators or to ensure that immune responses are self-limiting (29). Here, we have demonstrated that downregulation of CD16 on NK cells after cross-linking by IgG–Ag immune complexes is an example of such self-regulation, curtailing NK cell degranulation and limiting their ability to respond to exogenous cytokines by constraining expression of the high-affinity IL-2 receptor. Moreover, we have shown that CD16 downregulation is due to ADAM17-mediated shedding of CD16 from the NK cell surface, is induced *in vivo* after systemic vaccination, and is sustained for at least 12 weeks *in vivo* and at least 18 days *in vitro*. Taken together, these data suggest that tightly controlled surface expression of CD16 represents an important mechanism for regulating NK cell function *in vivo*.

It has previously been shown that broad spectrum matrix metalloprotease inhibitors allow recovery of CD16 expression in cytokine-maintained NK cells from HIV-1 infected individuals (16) and prevent downregulation of CD16 *in vitro* in response to cytokines or PMA (19, 20). We have confirmed these observations, revealing a very specific role for the ADAM17 class of proteases in this process, and extended them to show that inhibition of CD16 shedding can potentiate NK cell cytotoxic function, opening up potential therapeutic applications. However, TAPI-1 treatment enhanced degranulation more consistently than blockade with the ADAM-17-specific monoclonal antibody, suggesting that although reported as an ADAM-17 specific, this inhibitor may target additional MMPs involved in cytolytic granule processing (30, 31).

The prolonged period of CD16 downregulation *in vivo/ex vivo* after immunization (at least 3 months) was a surprise and raises questions about the ability of NK cells to mediate ADCC reactions in the immediate aftermath of vaccination or infection. Given that this is a global, generic effect rather than an antigen-specific effect, the consequences could be wide-ranging and might conceivably contribute to the increased risk of secondary infection after a primary viral infection, for example. Indeed, cross-linking of CD16 with rituximab (anti-CD20) has been shown to induce SHP-1-dependent hypo-responsiveness of NK cells to a diverse array of activating signals, including third-party tumor cell lines and cross-linking of NKp46, 2B4, NKG2D, and DNAM-1 (32). CD16 cross-linking and downregulation may, therefore, represent a dominant early pathway for controlling NK cell activation, affecting diverse NK cell signaling pathways. In line with this, we observed that CD16 downregulation also affects cytokine-mediated pathways of NK cell activation, and vice versa, raising further questions about the consequences of this homeostatic mechanism. It suggests, for example, that CD4+ T cell/IL-2-mediated activation of NK cells (3, 23, 27) may be unable to fully compensate for the inhibition of the ADDC pathway. On the other hand, CD25 expression by CD57^−^ NK cells – which are the major cytokine-producing subset of NK cells (27, 33) – was less affected by loss of CD16 (by comparison with CD57^+^ cells), indicating that cytokine responsiveness may be retained by a subset of the NK cell population despite widespread downregulation of CD16.

Persistence of CD16^low^ NK cells has been reported in chronically HIV-1 infected individuals despite effective suppression of viral replication by anti-retroviral therapy (16), but persistence of antigen – and, thus, of immune complexes – cannot be ruled out in this case. While long-term persistence of antigen cannot be entirely ruled out in our TIV model, the vaccine is inactivated. On the other hand, our data do imply that individual NK cells may have very limited capacity to re-express CD16 once it has been lost: NK cells that had been induced to shed CD16 showed very little propensity to re-express CD16 over a period of more than 2 weeks in culture, despite the absence of IgG or antigen. Recovery of the CD16^+^ NK cell population *in vivo* may, thus, rely on repopulation of the periphery from NK cell precursors.

Finally, our study raises questions about the nature of protection induced by influenza vaccination. The very low levels of circulating IgG induced by the live attenuated intra-nasal vaccine, and the correspondingly low levels of NK cell degranulation/ADCC/CD16 shedding, suggest that NK cell ADCC to influenza is not potentiated by LAIV. Anti-influenza IgG antibodies not only mediate NK cell ADCC but are sufficient to control virulent (H1N1 pandemic) infection in rhesus macaques in the absence of neutralizing antibodies (34, 35). It is interesting to speculate, therefore, that the lack of this ADCC response may in part explain the apparently much lower efficacy of LAIV compared to TIV that is now being recognized (CDC Advisory Committee on Immunization Practices, http://www.cdc.gov/media/releases/2016/s0622-laiv-flu.html).

## Author Contributions

MG directed research, designed the study, designed and performed experiments, analysed and interpreted data, and wrote the manuscript. CL designed and performed antibody determinations and antibody-dependent NK cell assays, and analyzed data. SS designed and performed the imagestream experiments and anayzed the data. AR-G designed and performed *ex vivo* and *in vitro* vaccine experiments and analyzed data. RB designed vaccination protocol, performed seasonal influenza vaccinations, and reviewed data. ER directed research and wrote the manuscript.

## Conflict of Interest Statement

The authors declare that the research was conducted in the absence of any commercial or financial relationships that could be construed as a potential conflict of interest.

## Supplementary Material

Figure S1 **Sensitivity of CD57-defined NK cell subsets to antigen–antibody-mediated downregulation of CD16**. Effect of TIV plus varying concentrations of immune plasma on CD16 (MFI) **(A,B)** and CD107a (%) **(C,D)** expression on CD56^bright^, CD56^dim^CD57^−^, CD56^dim^CD57^+^ subsets **(A,C)** and on CD57^+^NKG2C^−^, and CD57^+^NKG2C^+^ NK cells **(B,D)**. Paired comparisons were made between CD56dimCD57^−^ and CD56dimCD57 + NK cell subsets using Mann–Whitney *U* test. **p* < 0.05, ***p* < 0.01, ****p* < 0.001.

Figure S2 **The impact of MMP inhibition and ADAM17 blockade on early CD69 and CD25 expression**. PBMCs were cultured for 6 h with TIV and FCS or immune AB plasma (AB) in the presence or absence of the MMP inhibitor TAP1-1 **(A,C)** or the D1(A12) blocking antibody to ADAM17 **(B,D)** and the relevant negative controls (DMSO and mIgG1, respectively). CD69 (%) **(A,B)** and CD25 (%) **(C,D)** expression was assessed by flow cytometry on CD56^dim^, CD56^dim^CD57^−^, and CD56^dim^CD57^+^ NK cells. Data are presented for 10 different individuals. Paired comparisons between conditions were made using Mann–Whitney *U* test. **p* < 0.05, ***p* < 0.01, ****p* < 0.001.

## Abbreviations

ADCC, antibody-dependent cellular cytotoxicity; AEU, arbitrary ELISA units; HCC, high concentration of cytokines; NK, natural killer; TIV, trivalent influenza vaccine.
