# Supplementary figures and images for: Sustained Immune Complex-Mediated Reduction in CD16 Expression after Vaccination Regulates NK Cell Function
Source: Front Immunol. 2016 Sep 26;7:384. doi: 10.3389/fimmu.2016.00384 (PMC5035824; doi:10.3389/fimmu.2016.00384)

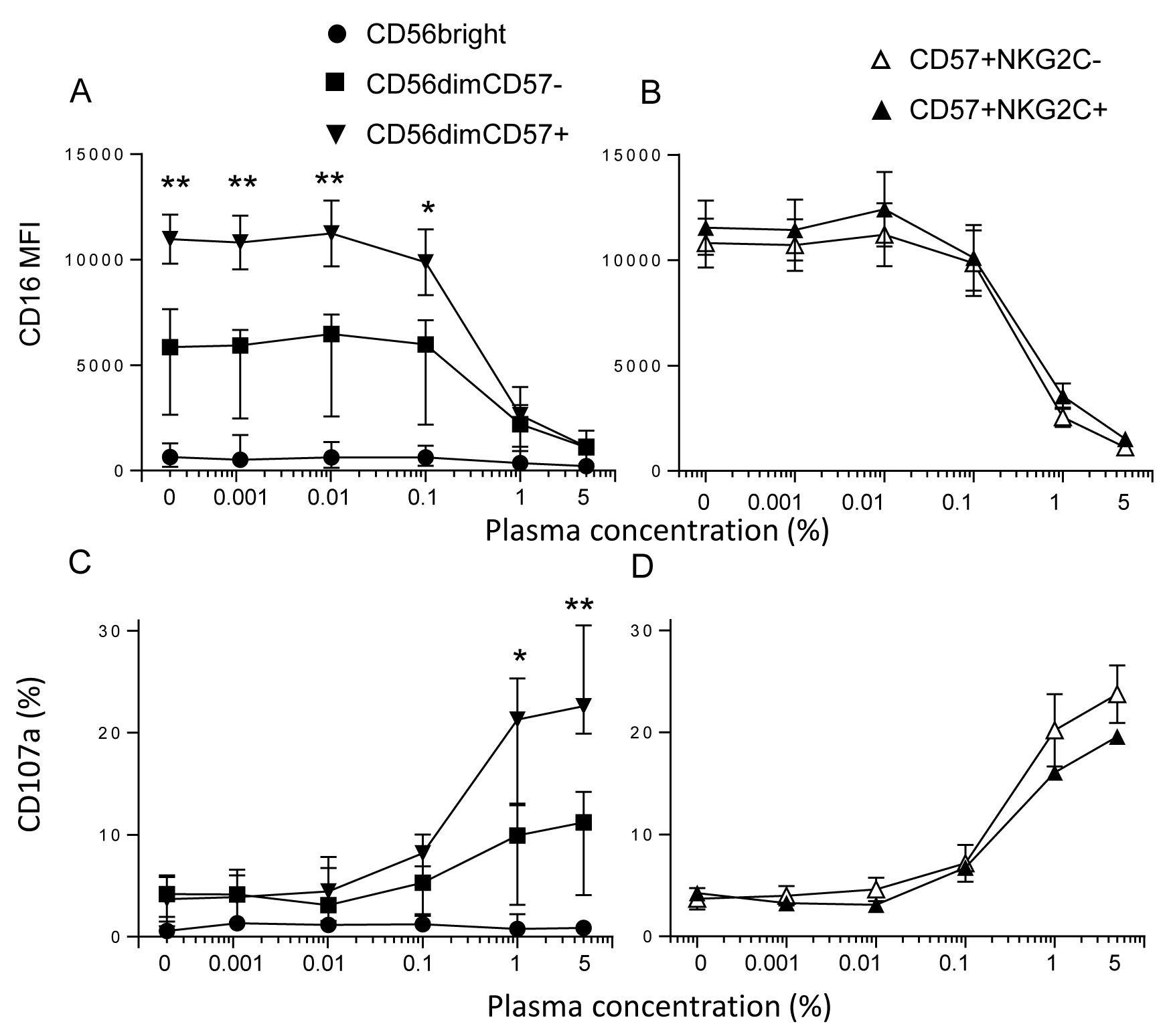

Supplement: Figure S1 — Sensitivity of CD57-defined NK cell subsets to antigen–antibody-mediated downregulation of CD16. Effect of TIV plus varying concentrations of immune plasma on CD16 (MFI) (A,B) and CD107a (%) (C,D) expression on CD56bright, CD56dimCD57−, CD56dimCD57+ subsets (A,C) and on CD57+NKG2C−, and CD57+NKG2C+ NK cells (B,D). Paired comparisons were made between CD56dimCD57− and CD56dimCD57 + NK cell subsets using Mann–Whitney U test. *p < 0.05, **p < 0.01, ***p < 0.001. [file image_1.tif]

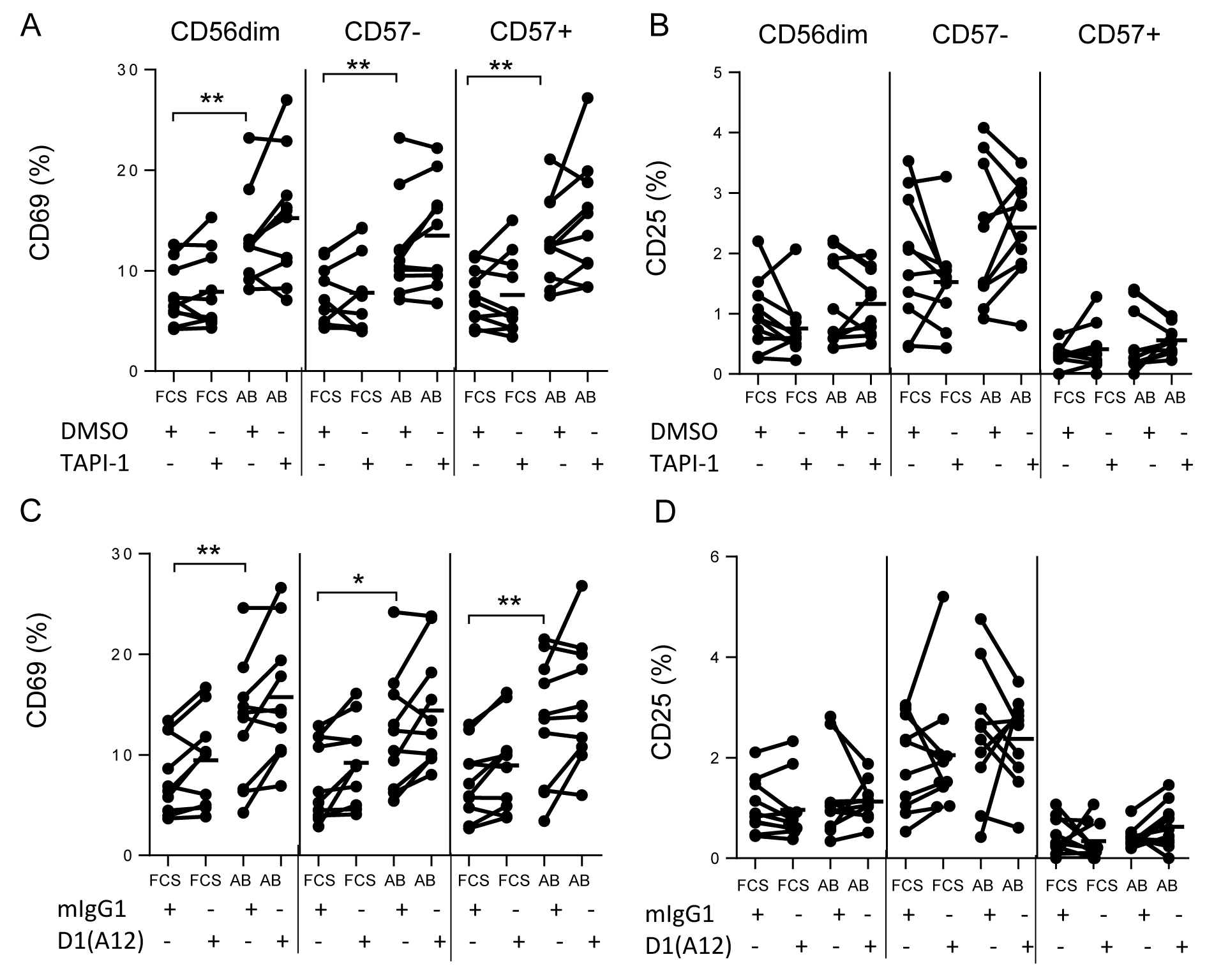

Supplement: Figure S2 — The impact of MMP inhibition and ADAM17 blockade on early CD69 and CD25 expression. PBMCs were cultured for 6 h with TIV and FCS or immune AB plasma (AB) in the presence or absence of the MMP inhibitor TAP1-1 (A,C) or the D1(A12) blocking antibody to ADAM17 (B,D) and the relevant negative controls (DMSO and mIgG1, respectively). CD69 (%) (A,B) and CD25 (%) (C,D) expression was assessed by flow cytometry on CD56dim, CD56dimCD57−, and CD56dimCD57+ NK cells. Data are presented for 10 different individuals. Paired comparisons between conditions were made using Mann–Whitney U test. *p < 0.05, **p < 0.01, ***p < 0.001. [file image_2.tif]
